# Using COVID-19 Vaccine Attitudes on Twitter to Improve Vaccine Uptake Forecast Models in the United States: Infodemiology Study of Tweets

**DOI:** 10.2196/43703

**Published:** 2023-08-21

**Authors:** Nekabari Sigalo, Naman Awasthi, Saad Mohammad Abrar, Vanessa Frias-Martinez

**Affiliations:** 1 College of Information Studies University of Maryland College Park, MD United States; 2 Department of Computer Science University of Maryland College Park, MD United States

**Keywords:** social media, Twitter, COVID-19, vaccine, surveys, SARS-CoV-2, vaccinations, hesitancy, vaccine hesitancy, forecast model, vaccine uptake, health promotion, infodemiology, health information, misinformation

## Abstract

**Background:**

Since the onset of the COVID-19 pandemic, there has been a global effort to develop vaccines that protect against COVID-19. Individuals who are fully vaccinated are far less likely to contract and therefore transmit the virus to others. Researchers have found that the internet and social media both play a role in shaping personal choices about vaccinations.

**Objective:**

This study aims to determine whether supplementing COVID-19 vaccine uptake forecast models with the attitudes found in tweets improves over baseline models that only use historical vaccination data.

**Methods:**

Daily COVID-19 vaccination data at the county level was collected for the January 2021 to May 2021 study period. Twitter’s streaming application programming interface was used to collect COVID-19 vaccine tweets during this same period. Several autoregressive integrated moving average models were executed to predict the vaccine uptake rate using only historical data (baseline autoregressive integrated moving average) and individual Twitter-derived features (autoregressive integrated moving average exogenous variable model).

**Results:**

In this study, we found that supplementing baseline forecast models with both historical vaccination data and COVID-19 vaccine attitudes found in tweets reduced root mean square error by as much as 83%.

**Conclusions:**

Developing a predictive tool for vaccination uptake in the United States will empower public health researchers and decisionmakers to design targeted vaccination campaigns in hopes of achieving the vaccination threshold required for the United States to reach widespread population protection.

## Introduction

### Background

Since the onset of the COVID-19 pandemic, there has been a global effort to develop vaccines that protect against COVID-19. Individuals who are fully vaccinated are far less likely to contract and therefore transmit the virus to others [1]. Up until recently, public health experts have stressed the importance of achieving a numerical threshold of herd immunity, but this is only possible if a significant proportion of the population is fully vaccinated. More recent research suggests that the traditional concept of herd immunity may not apply to COVID-19 [2]. Instead, the goal is to increase vaccination uptake to optimize population protection without prohibitive restrictions on our daily lives [3]. Accurately forecasting vaccination uptake allows policymakers and researchers to evaluate how close we are to achieving normalcy again.

Researchers have turned to traditional methods for forecasting COVID-19 infection and vaccination rates [4-6]. For example, one of the most common forecasting methods used, univariate time series, involves predicting future vaccination rates using historical vaccination rates. While this method can be useful in many cases, it fails to account for other time-dependent factors that may also influence vaccinations. For example, the COVID-19 vaccine conversation on social media has been deemed an infodemic, with antivaccination misinformation spreading across social media platforms [7]. Researchers have found that the internet and social media both play a role in shaping personal or parental choices about vaccinations [8,9]. Additionally, previous research showed a positive relationship between positive sentiment scores in COVID-19 vaccine–related tweets and an increase in vaccination rates [10]. These findings suggest it is important to consider the daily conversations on social media when developing vaccine uptake forecast models.

### Forecasting COVID-19–Related Measures Using Social Media

There is no shortage of studies that sought to forecast COVID-19-related measures using information from social media. Researchers Yousefinaghani et al [11] conducted a study using COVID-19–related terms mentioned in tweets and Google searches to predict COVID-19 waves in the United States. Researchers found that tweets that mentioned COVID-19 symptoms predicted 100% of first waves of COVID-19 days sooner than other data sources. Another study used data from Google searches, tweets, and Wikipedia page views to predict COVID-19 cases and deaths in the United States [12]. Researchers found models that included features from all 3 sources performed better than baseline models that did not include these features. Researchers also found that Google searches were a leading indicator of the number of cases and deaths across the United States. Another study [13] examined the relationship between daily COVID-19 cases and COVID-19–related tweets and Google Trends. In a study conducted by Shen et al [14], researchers used reports of symptoms and diagnoses on Weibo, a popular social media platform in China, in order to predict COVID-19 case counts in mainland China. Researchers found reports of symptoms and diagnoses on the social media platform to be highly predictive of daily case counts. Although each of these studies forecast COVID-19 cases and deaths, none of these studies forecast COVID-19 vaccination rates.

### Forecasting Vaccinations

Very few studies have conducted time series forecasting of the COVID-19 vaccinated population in the United States. In a study conducted by Sattar and Arifuzzaman [15], researchers developed a time series model to predict the percentage of the US population that would get at least 1 dose of the COVID-19 vaccine or be fully vaccinated. Researchers projected that by the end of July 2021, 62.44% and 48% of the US population would get at least 1 dose of the COVID-19 vaccine or be fully vaccinated, respectively. Although this paper also included a separate tweet sentiment analysis, researchers did not include Twitter-related features in the forecast model. Additionally, researchers used aggregated vaccination data for the entire United States, rather than a more granular geographic level.

Another study aimed to evaluate if and when the world would reach a vaccination rate sufficient enough for herd immunity by forecasting the number of people fully vaccinated against COVID-19 in various countries, including the United States [16]. In this study, researchers used a common univariate time series forecasting method, autoregressive integrated moving average (ARIMA), to forecast the future number of fully vaccinated people using only historical vaccination data. Based on the resulting projections, researchers concluded that countries were nowhere near the necessary herd immunity threshold needed to end the COVID-19 pandemic.

A study conducted by Cheong et al [17] sought to predict COVID-19 vaccine uptake using various sociodemographic factors. Although not a time series forecasting model, the results of this study showed that geographic location, education level, and online access were highly predictive of vaccination uptake in the United States. The model predicted vaccine uptake with 62% accuracy.

Although there are very few studies related to COVID-19 vaccination forecasting, other studies have been conducted to predict immunizations for other illnesses. For example, 1 study analyzed electronic medical records of a cohort of 250,000 individuals over the course of 10 years [18]. Researchers developed a model to predict vaccination uptake of individuals in the upcoming influenza season based on previous personal and social behavioral patterns. Another study developed a tool for leveraging immunization related content from Twitter and Google Trends to develop a model for predicting whether a child would receive immunizations [19]. Researchers were able to predict child immunization statuses with 76% accuracy.

### Study Objectives

Although previous studies have developed forecast models for COVID-19 vaccination rates in the United States, to our knowledge, there are no studies that aim to factor in the real-time vaccination attitudes present on Twitter. The vaccine attitudes on Twitter change daily, as do vaccination rates, so analyzing vaccine attitudes on social media might contribute to the performance of vaccine forecast models. Additionally, previous studies developed forecast models that focused on the entire United States as a whole. These forecast models fail to appreciate the differences in vaccination roll out, behaviors, and attitudes across different geographic regions. This study seeks to fill this gap by examining vaccine uptake at the metropolitan level.

The purpose of this study is to develop a time series forecasting algorithm that can predict future vaccination rates across US metropolitan areas. Specifically, this study aims to determine whether supplementing forecast models with real-time vaccine attitudes found in tweets—measured via sentiments and emotions—improves over baseline models that only use historical vaccination data. Developing a predictive tool for vaccination uptake in the United States will empower public health researchers and decision makers to design targeted vaccination campaigns in hopes of achieving the vaccination threshold required for us to reach herd immunity.

## Methods

### Data Collection and Preprocessing

#### Twitter Data

The Twitter streaming application programming interface, which provides access to a random sample of 1% of publicly available tweets, was used to collect tweets from 8 of the most populated metropolitan areas in the United States from January 2021 to May 2021 (Textbox 1) [20]. We chose to focus on large metropolitan areas to gather a sufficient number of tweets for the analysis. Additionally, larger metropolitan areas also tend to have users who enable the location feature when tweeting [21,22]. All tweets had “place” information (usually city and state). The place information found in tweets was used to determine the metropolitan area associated with each tweet. Next, to extract tweets related to COVID-19 vaccines, tweets were further filtered by matching variations of vaccine-related keywords, such as *vaccine, pfizer, moderna, johnson & johnson, and dose*. Additional vaccine keywords can be found in Multimedia Appendix 1. A language filter was then applied to identify tweets written in the English language. The tweets sample was further preprocessed to minimize “noise” resulting from tweets that matched our vaccine-related keywords but did not necessarily reflect the thoughts and opinions of individual Twitter users. For example, companies often promote job postings and advertisements on Twitter using targeted hashtags in hopes of reaching their target audience. To prevent these tweets from adding noise to the sample, tweets related to job postings and advertisements were removed by excluding tweets with hashtags and keywords, including “jobs,” “hiring,” “advertisement,” “apply,” and “ad.”

**Targeted metropolitan areas for Twitter data collection, January 1, 2021, to May 20, 2021.**

- Phoenix-Mesa-Chandler, AZ
- Miami–Fort Lauderdale–Pompano Beach, FL
- Atlanta–Sandy Springs–Alpharetta, GA
- New York–Newark–Jersey City, NY-NJ-PA
- Philadelphia-Camden-Wilmington, PA-NJ-DE-MD
- Washington-Arlington-Alexandria, DC-VA-MD-WV
- Chicago-Naperville-Elgin, IL-IN-WI
- Los Angeles–Long Beach–Anaheim, CA

#### COVID-19 Vaccination Data

Daily COVID-19 vaccination data at the county level was collected for the January 2021 to May 2021 study period from the Centers for Disease Control and Prevention’s publicly available vaccination data set [23]. This data set includes daily vaccination data from clinics, pharmacies, long-term care facilities, dialysis centers, Federal Emergency Management Agency and Health Resources and Services Administration partner sites, and federal entity facilities. Vaccination administration data are reported to the Centers for Disease Control and Prevention via immunization information systems, the vaccine administration management system, and data submissions directly to the COVID-19 Data Clearinghouse [23]. Each county was linked to its respective metropolitan area according to the US Census delineation file [24]. Next, the data were aggregated to the daily-metropolitan level and the 7-day rolling average of the percentage of individuals who have been administered at least 1 vaccine dose was calculated.

### Data Analysis

#### Sentiment and Emotion Analysis of Tweets

For the purposes of this study, we measure COVID-19 vaccine attitudes via sentiment and emotion analyses of tweets. We evaluated both sentiments and emotions because both methods offer different levels of granularity. Sentiment analysis focuses on determining the overall sentiment or polarity of a text, such as positive, negative, or neutral. It provides a high-level understanding of the sentiment expressed. Emotion analysis, on the other hand, aims to identify specific emotions within the text, such as joy, anger, and sadness. It offers a more detailed and nuanced understanding of the emotional states. By utilizing both sentiment and emotion analysis, we gain a comprehensive understanding of the text, covering both the overall sentiment and the specific emotions expressed.

To capture the sentiments and emotions found in COVID-19 vaccine-related tweets, a sentiment and emotion analysis of all tweets was conducted using bidirectional encoder representation from transformer (BERT) [25], a pretrained language model trained using bidirectional (left to right and right to left) context training to learn joint probability distributions of text. We leveraged the fine-tuned BERT models in the *TweetNLP* package in Python (Python Software Foundation) [26] to calculate the valence of 8 different emotions (fear, joy, anticipation, anger, disgust, sadness, surprise, trust), along with overall neutral, positive, and negative sentiment of tweets in our analysis sample. The sentiment analysis and emotion recognition BERT models were fine-tuned with the TweetEval benchmark [27].

The outputs from BERT are softmax of logits, one corresponding to each of the emotions or sentiments. For each tweet, we performed argmax over the probability distribution for each tweet, to get the most likely emotion and sentiment. Next, we found the percentage of tweets classified as each of the emotions and sentiments for each day and metro area combination. For example, the count of anger tweets on January 1 for the New York–Newark–Jersey City, NY-NJ-PA metropolitan area divided by the total number of tweets on January 1 for the New York–Newark–Jersey City, NY-NJ-PA metropolitan area gives percentage of anger tweets for January 1 in the New York–Newark–Jersey City, NY-NJ-PA metropolitan area.

The total number of COVID-19 vaccine related tweets and users per 100,000 population was also calculated for each day of data collection, at the metropolitan level. Finally, user engagement metrics, including the average number of retweets and favorites, were calculated for each day of data collection, at the metropolitan level. Retweets and favorites suggest, after processing the information, that a user resonates with an idea expressed in a tweet [28,29]. Therefore, we believe these engagement metrics might also reflect vaccine attitudes.

#### Time Series Model

The data were divided into training and test data sets, where the time series analysis was trained using the data set created from the January 1, 2021, to April 12, 2021, time period, and tested on the data set created from the April 13, 2021, to May 20, 2021, time period. ARIMA models were executed for forecasting the proportion of individuals who have been administered at least 1 vaccine dose. Autoregressive integrated moving average exogenous variable model (ARIMAX) models, which are extensions of ARIMA models that include independent predictors called exogenous variables, were also executed. The ARIMA method has been widely used in time series forecasting and public health surveillance [30-32]. An ARIMA model typically consists of three components: (1) auto-regression, notated in the model as *p*; (2) differencing, notated in the model as *d*; and (3) moving average, notated in the model as *q* [33]. In an ARIMA model, the present value of the time-series is a linear function of random noise and its previous values; the present value is also a linear function of both present and past values of the residuals in the model; and the auto-regressive moving average model includes both the auto-regressive and moving average models, in addition to the historical values in the time series and its residuals [30].

Stationarity of a time series is a key assumption when making predictions based on past observations of a variable [34]. Stationarity requires the properties (mean and variance) of a time series to remain constant over time, thus making future values easier to predict [35]. Otherwise, the results are spurious and analyses are not valid [30]. The stationarity of all variables included in the time series was assessed using the Dickey-Fuller (dfuller) test. If the null hypothesis is rejected, stationarity is satisfied. If stationarity is not satisfied, variables must undergo differencing, a process that removes any trend in the times series that is not of interest [35]. All differencing and model selection was performed by the auto_arima function from the *pmdarima* package in Python [36], which is a function that selects the optimal order of the model based on the Hyndman-Khandakar algorithm for automatic ARIMA modeling [37]. A combination of unit root tests and minimization of the Akaike information criterion and Bayesian information criterion allows this algorithm to select the best preforming model order by fitting several variations of model components *p, d,* and *q* [38]. By including a penalty that is an increasing function of the number of estimated parameters, the information criteria scores maximize the goodness of fit while minimizing the number of model parameters, effectively dealing with both the risk of overfitting and the risk of underfitting [39,40].

For each metropolitan area, a baseline ARIMA model with no exogenous variables was constructed to forecast the 7-day rolling average of the number of individuals who have been administered at least 1 vaccine dose, using only past values of this outcome. To assess the ability of vaccine attitudes on Twitter to improve COVID-19 vaccination forecasts, multiple ARIMAX models were executed, each with individual Twitter-derived features included as exogenous variables. Additionally, we executed a multivariate ARIMAX model that included those Twitter attitudes that showed improvement over the ARIMA baseline across all metro areas. A final ARIMAX model that contained all Twitter features regardless of performance was attempted but did not converge. A complete list of the constructed time series models can be found in Table 1.

**Table 1:** Time series models predicting COVID-19 vaccine uptake, January 1, 2021, to May 20, 2021.

Model type	Exogenous variables
ARIMA^a^	None (baseline)
ARIMAX^b^	Number of users per 100,000 population
ARIMAX	Number of tweets per 100,000 population
ARIMAX	Average favorites
ARIMAX	Average retweets
ARIMAX	% Positive sentiment
ARIMAX	% Negative sentiment
ARIMAX	% Neutral sentiment
ARIMAX	% Trust
ARIMAX	% Surprise
ARIMAX	% Sadness
ARIMAX	% Joy
ARIMAX	% Fear
ARIMAX	% Disgust
ARIMAX	% Anticipation
ARIMAX	% Anger
ARIMAX	Best predictors (predictors that show improvement over baseline across all metro areas)

^a^ARIMA: autoregressive integrated moving average.

^b^ARIMAX: autoregressive integrated moving average exogenous variable model.

### Ethical Considerations

This project does not meet the definition of human participant research under the purview of the University of Maryland Institutional Review Board according to federal regulations, section 45CFR46.102(e) [41].

## Results

### Twitter Data

A total of 59,687 COVID-19 vaccine-related tweets were collected during the data collection period, across 23,878 users (Table 2). The Los Angeles–Long Beach–Anaheim metropolitan area had the largest representation of tweets (13,125/59,687, 21.99%) as well as the largest representation of users (5620/23,878, 23.54%). The Miami–Fort Lauderdale–Pompano Beach metropolitan area had the smallest representation of tweets (1631/59,687, 2.73%) as well as the smallest representation of users (625/23,878, 2.62%). The maximum number of tweets by a single individual was 228 (from a user in the Washington-Arlington-Alexandria metropolitan area).

**Figure 1:**
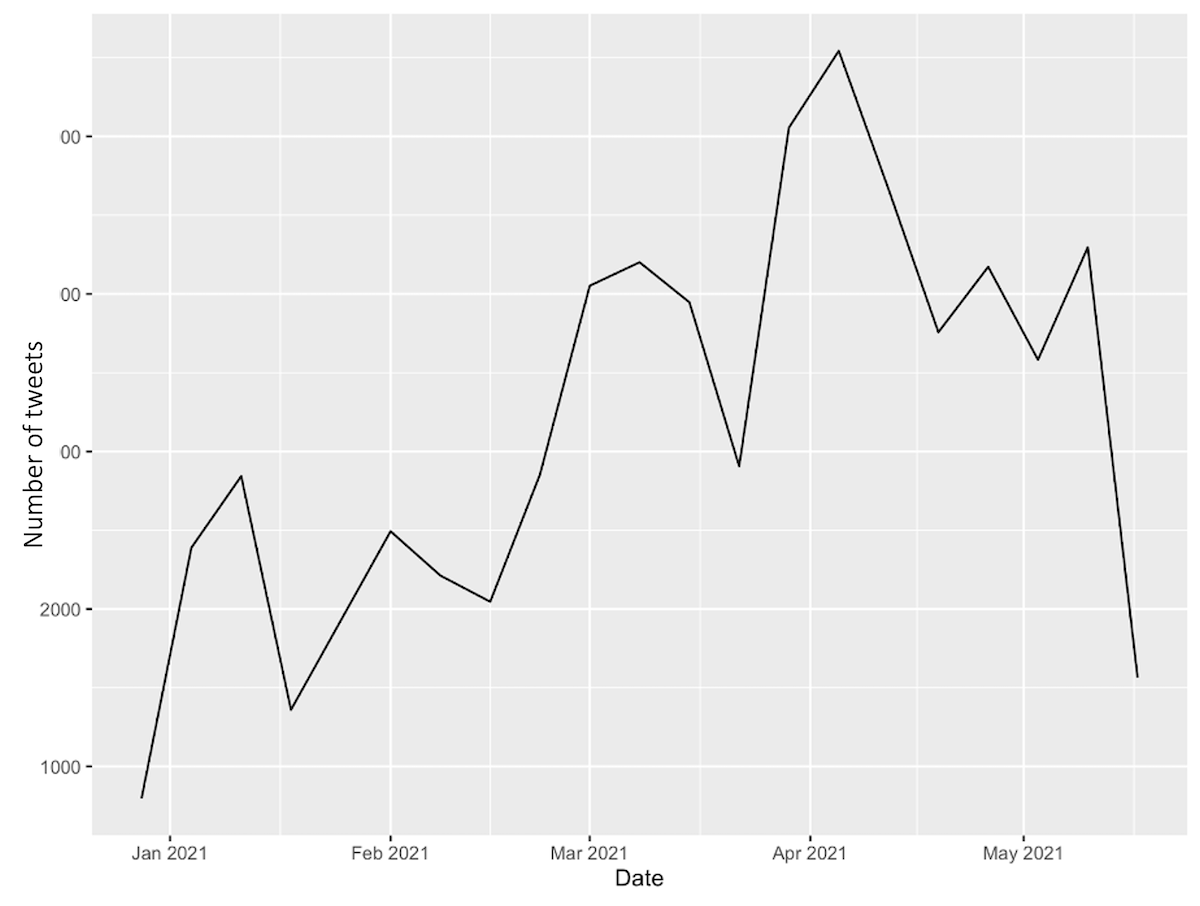
Number of COVID-19 vaccine tweets over time, across all metropolitan areas, January 1, 2021, to May 20, 2021.

The temporal trends for the number of COVID-19 vaccine–related tweets from January to May 2021 are presented in Figure 1. The number of COVID-19 vaccine–related tweets fluctuated over time; however, a peak in the number of tweets was observed during the week of April 5, 2021, to April 11, 2021. This was the week that President Joe Biden announced that every adult in the United States would be eligible to receive a COVID-19 vaccine starting April 19, 2021 [42].

**Table 2:** Number of COVID-19 vaccine tweets (n=59,687) and users (n=23,878) by city, January 1, 2021, to May 20, 2021.

Metropolitan area	Tweets, n, %	Users, n, %	Average retweets, mean (SD)	Average favorites, mean (SD)
Atlanta–Sandy Springs–Alpharetta, GA	12,623 (21.1)	5431 (22.7)	438 (5140)	10 (178)
Chicago-Naperville-Elgin, IL-IN-WI	6857 (11.5)	2847 (11.9)	543 (9579)	11 (118)
Los Angeles–Long Beach–Anaheim, CA	12,387 (20.8)	4858 (20.3)	351 (4209)	13 (224)
Miami–Fort Lauderdale–Pompano Beach, FL	4345 (7.3)	1558 (6.5)	267 (3187)	131 (2389)
New York–Newark–Jersey City, NY-NJ-PA	2231 (3.7)	914 (3.8)	169 (1704)	6 (20)
Philadelphia-Camden-Wilmington, PA-NJ-DE-MD	6488 (10.9)	2025 (8.5)	304 (3952)	13 (124)
Phoenix-Mesa-Chandler, AZ	12,623 (21.1)	5431 (22.7)	438 (5140)	10 (178)
Washington-Arlington-Alexandria, DC-VA-MD-WV	6857 (11.5)	2847 (11.9)	543 (9579)	11 (118)

### Sentiment and Emotion Analysis

A sentiment analysis classified most tweets across all metropolitan areas as having neutral sentiment, with joy as the predominantly expressed emotion (Table 3). The Phoenix-Mesa-Chandler metropolitan area had the largest proportion of tweets with positive sentiment (3875/12,623, 30.7%), while the Miami–Fort Lauderdale–Pompano Beach metropolitan area had the lowest proportion of tweets with positive sentiment (1065/4345, 24.5%). Anger and disgust were the most perceived negative emotions. The Atlanta–Sandy Springs–Alpharetta, GA metropolitan area had the largest proportion of tweets with negative sentiment (3888/12,623, 30.8%), while the Miami–Fort Lauderdale–Pompano Beach metropolitan area had the lowest proportion of tweets with negative sentiment (1060/4345, 24.4%).

**Table 3:** Distribution of sentiments and emotions among COVID-19 vaccine tweets collected from January 1, 2021, to May 20, 2021 (N=59,687).

Metropolitan area	Anger, %	Anticipation, %	Disgust, %	Fear, %	Joy, %	Sadness, %	Surprise, %	Trust, %	Negative, %	Neutral, %	Positive, %
Atlanta–Sandy Springs–Alpharetta, GA	18.1	24	14.2	7	30.4	6	0.2	0	30.8	42	27.2
Chicago-Naperville-Elgin, IL-IN-WI	16.9	23.4	13.9	6.3	33.7	5.7	0.1	0.1	29.1	40.4	30.6
Los Angeles–Long Beach–Anaheim, CA	17.4	22.4	12.7	7.1	33.4	6.9	0.2	0	30.4	39.9	29.7
Miami–Fort Lauderdale–Pompano Beach, FL	14.2	28.1	13.9	5.6	32.4	5.5	0.1	0.1	24.4	51.1	24.5
New York–Newark–Jersey City, NY-NJ-PA	17.5	24.9	13.7	6.5	31.8	5.3	0.2	0.1	28.8	42.3	28.9
Philadelphia-Camden-Wilmington, PA-NJ-DE-MD	18.5	26	13.4	6.9	29.2	5.8	0.1	0	28.6	44.1	27.4
Phoenix-Mesa-Chandler, AZ	18	23	13.9	7.1	32.2	5.7	0.1	0	28.5	40.8	30.7
Washington-Arlington-Alexandria, DC-VA-MD-WV	15.3	28.6	14.3	6.8	29.6	5.3	0.1	0	28.2	44.7	27.1

### Time Series Forecast

Multiple time series models were constructed to forecast the vaccine uptake rate (7-day rolling average). The results of the Dickey-Fuller (dfuller) test for stationarity revealed that across all metropolitan areas, stationarity did not hold for several of the variables (Tables 4 and 5). However, the necessary differencing was automatically applied via the auto_arima function.

The performance of the optimal models across all regions, as determined by the auto_arima function, can be found in Tables 6 and 7. The best-performing model for each metropolitan area is marked by an asterisk. Models that performed better than the baseline model are bolded. Model performance for the “out-sample” forecasts was evaluated using the root mean square error (RMSE) instead of Akaike information criterion because RMSE measures how close the data are around the line of best fit [43]. This measure is commonly used in time series forecasting to evaluate how close the forecasted values are to the actual values [44]. When evaluating model performance using RMSE, across all metropolitan areas, the addition of a Twitter-derived feature related to COVID-19 vaccination attitudes improved model performance by up to 83%. For example, across all metropolitan areas, adding the *percentage of vaccine tweets expressing joy, negative sentiment, surprise, or trust* individually as exogenous variables resulted in a lower RMSE compared to the baseline ARIMA model. Additionally, across all metropolitan areas, most of the ARIMAX models, which each had 1 Twitter-derived feature related to COVID-19 vaccination attitudes, showed improvement over the baseline ARIMA model that did not factor in Twitter-derived features. A final model that contained the 3 features that consistently showed improvement over baseline across all metro areas (negative sentiment [%], surprise [%], joy [%], trust [%]) showed improvement over the baseline ARIMA when combined into 1 model (ARIMAX with multiple exogenous variables) across all metropolitan areas except for Philadelphia-Camden-Wilmington, PA-NJ-DE-MD and Phoenix-Mesa-Chandler, AZ.

**Table 4:** Dickey-Fuller (dfuller) test for stationarity in Atlanta–Sandy Springs–Alpharetta, GA, Chicago-Naperville-Elgin, IL-IN-WI Los Angeles-–Long Beach–Anaheim, CA, and Miami–Fort Lauderdale-–Pompano Beach, FL.

Variable	Atlanta–Sandy Springs–Alpharetta, GA		Chicago-Naperville-Elgin, IL-IN-WI		Los Angeles–Long Beach–Anaheim, CA		Miami–Fort Lauderdale–Pompano Beach, FL	
	Test statistic	*P* value	Test statistic	*P* value	Test statistic	*P* value	Test statistic	*P* value
% Anger	−1.237	.66^a^	−3.091	.03	−2.512	.11^a^	−3.776	.003
% Anticipation	−1.879	.34^a^	−2.579	.10^a^	−1.594	.49^a^	−3.375	.01
Average favorites	−4.154	.001	−3.073	.03	−4.080	.001	−2.956	.04
Average retweets	−2.882	.047	1.632	.99^a^	−3.526	.007	−3.239	.02
% Disgust	−2.915	.04	−2.435	.13^a^	−1.711	.42^a^	−3.414	.01
% Fear	−2.908	.04	−3.698	.004	−3.195	.02	−2.707	.07^a^
% Joy	−1.548	.51^a^	−2.500	.12^a^	−1.354	.60^a^	−2.264	.18^a^
% Negative sentiment	−1.666	.45^a^	−2.198	.21^a^	−1.425	.57^a^	−2.142	.23^a^
% Neutral sentiment	−2.223	.20^a^	−3.820	.003	−1.655	.46^a^	−2.841	.05^a^
Number of tweets per 100,000 population	−1.521	.52^a^	−1.333	.61^a^	−1.221	.66^a^	−2.001	.29^a^
Number of users per 100,000 population	−1.450	.56^a^	−1.334	.61^a^	−1.241	.66^a^	−1.947	.31^a^
% Positive sentiment	−1.281	.64^a^	−2.626	.09^a^	−1.256	.65^a^	−2.847	.05^a^
Percentage of individuals who have been administered at least 1 vaccine dose (7-day rolling average)	−0.569	.88^a^	−0.814	.82^a^	−0.048	.95^a^	0.057	.96^a^
% Sadness	−3.817	.003	−2.619	.09^a^	−3.157	.02	−3.249	.02
% Surprise	−4.030	.001	−2.349	.16^a^	−3.883	.002	−1.658	.45^a^
% Trust	−2.739	.07^a^	−3.128	.02	−2.120	.24^a^	−5.039	<.001

^a^Nonstationary variable results.

**Table 5:** Dickey-Fuller (dfuller) test for stationarity in New York–Newark–Jersey City, NY-NJ-PA, Philadelphia-Camden-Wilmington, PA-NJ-DE-MD, Phoenix-Mesa-Chandler, AZ, and Washington-Arlington-Alexandria, DC-VA-MD-WV.

	New York–Newark–Jersey City, NY-NJ-PA		Philadelphia-Camden-Wilmington, PA-NJ-DE-MD		Phoenix-Mesa-Chandler, AZ		Washington-Arlington-Alexandria, DC-VA-MD-WV	
Variable	Test statistic	*P* value	Test statistic	*P* value	Test statistic	*P* value	Test statistic	*P* value
% Anger	−3.084	.03	−4.880	<.001	−3.275	.02	−2.233	.20^a^
% Anticipation	−3.336	.01	−3.586	.006	−3.400	.01	−2.111	.24^a^
Average favorites	−2.786	.06^a^	−3.001	.04	−3.367	.01	−2.507	.11^a^
Average retweets	−2.724	.07^a^	−1.647	.46^a^	−2.596	.10^a^	−2.451	.13^a^
% Disgust	−2.218	.20^a^	−2.307	.17^a^	−1.678	.44^a^	−2.330	.16^a^
% Fear	−2.730	.07^a^	−3.129	.02	−3.625	.005	−2.960	.043
% Joy	−2.383	.15^a^	−2.702	.07^a^	−1.826	.37^a^	−1.493	.54^a^
% Negative sentiment	−1.432	.57^a^	−2.897	.046	−1.846	.36^a^	−1.747	.41^a^
% Neutral sentiment	−1.993	.29^a^	−3.635	.005	−1.738	.41^a^	−1.998	.29^a^
Number of tweets per 100,000 population	−1.402	.58^a^	−1.617	.47^a^	−1.205	.67^a^	−1.890	.34^a^
Number of users per 100,000 population	−1.461	.55^a^	−1.697	.43^a^	−1.116	.71^a^	−1.796	.38^a^
% Positive sentiment	−1.702	.43^a^	−3.793	.003	−2.080	.25^a^	−1.572	.498^a^
Percentage of individuals who have been administered at least 1 vaccine dose (7 day rolling average)	−0.792	.82^a^	−1.064	.73^a^	−1.483	.54^a^	−0.085	.95^a^
% Sadness	−2.862	.05	−2.263	.18^a^	−3.206	.02	−1.954	.31^a^
% Surprise	−2.893	.046	−2.599	.09^a^	−3.082	.03	−1.544	.51^a^
% Trust	−2.733	.069^a^	−2.463	.06^a^	−2.854	.05^a^	−3.078	.03

^a^Nonstationary variable results.

**Table 6:** ARIMA^a^/ARIMAX^b^ model performance (RMSE^c^) for Atlanta–Sandy Springs–Alpharetta, GA, Chicago-Naperville-Elgin, IL-IN-WI, Los Angeles–Long Beach–Anaheim, CA, and Miami–Fort Lauderdale–Pompano Beach, FL. Models that performed better than the baseline ARIMA are shown in italics.

Variables	Atlanta–Sandy Springs–Alpharetta, GA, RMSE	Chicago-Naperville-Elgin, IL-IN-WI, RMSE	Los Angeles–Long Beach–Anaheim, CA, RMSE	Miami–Fort Lauderdale–Pompano Beach, FL, RMSE
(Baseline) percentage of individuals who have been administered at least 1 vaccine dose (7 day rolling average)	4.0855	4.2182	4.1473	3.7109
Number of users per 100,000 population	*1.6356*	*1.3900*	*0.7198*	*1.2971*
Number of tweets per 100,000 population	*1.6420*	*1.3700*	*0.7131*	*1.3492*
Average favorites	*2.1176*	*4.2100*	*0.6878*	4.0045
Average retweets	5.3545	*1.2414*	*4.1356*	*1.4062*
% Positive sentiment,	*1.6182*	*1.3000*	*0.7051*	*1.1691*
% Negative sentiment	*1.6238*	*1.3300*	*0.6915*	*1.2168*
% Neutral sentiment	*1.6236*	*1.1600* ^*d*^	*0.7213*	3.7217
% Trust	*4.0854*	4.2183	*4.1471*	*1.1407*
% Surprise	*1.6522*	*1.3371*	*0.7078*	*1.1314*
% Sadness	*1.5826* ^*d*^	*1.2400*	*0.7077*	3.7117
% Joy	*1.6243*	*1.3600*	*0.6865* ^*d*^	*1.2322*
% Fear	*1.6751*	*4.1900*	*0.6973*	*3.7028*
% Disgust	*1.6401*	*1.3200*	*0.7054*	3.7670
% Anger	4.6909	*4.2000*	*0.7037*	*1.1006* ^*d*^
% Anticipation	*1.6589*	*1.2800*	*0.7079*	3.7115
Best predictors: joy (%), negative sentiment (%), surprise (%), trust (%)	*1.7324*	*1.2878*	*0.6921*	*1.2901*

^a^ARIMA: autoregressive integrated moving average.

^b^ARIMAX: autoregressive integrated moving average exogenous variable model.

^c^RMSE: root mean square error.

^d^Best-performing model for each metropolitan area.

**Table 7:** ARIMA^a^/ARIMAX^b^ model performance (RMSE^c^) for New York–Newark–Jersey City, NY-NJ-PA, Philadelphia-Camden-Wilmington, PA-NJ-DE-MD, Phoenix-Mesa-Chandler, AZ, and Washington-Arlington-Alexandria, DC-VA-MD-WV. Models that performed better than the baseline ARIMA are shown in italics.

	New York–Newark–Jersey City, NY-NJ-PA, RMSE	Philadelphia-Camden-Wilmington, PA-NJ-DE-MD, RMSE	Phoenix-Mesa-Chandler, AZ, RMSE	Washington-Arlington-Alexandria, DC-VA-MD-WV, RMSE
(Baseline) percentage of individuals who have been administered at least 1 vaccine dose (7 day rolling average)	4.7686	4.4737	2.7743	2.5829
Number of users per 100,000 population	*1.8473*	*1.2524*	*1.9983*	*1.1554*
Number of tweets per 100,000 population	*1.8329*	*1.2737*	*1.9769*	*1.1259*
Average favorites	*4.7564*	5.6078	*1.9406*	*0.7570*
Average retweets	*1.8838*	*0.9200^d^*	*2.7728*	*0.7570*
% Positive sentiment,	*1.8682*	4.8339	*1.9452*	*1.1168*
% Negative sentiment	*1.8757*	*1.2486*	*1.9118*	*1.1206*
% Neutral sentiment	4.7722	*1.2392*	*1.8932*	2.5825
% Trust	*1.8659*	*1.2503*	*1.9372*	*1.1327*
% Surprise	*4.7668*	*1.2279*	*1.9374*	*1.1210*
% Sadness	*4.4896*	*1.1615*	*1.9355*	*2.4392*
% Joy	*1.8397*	*1.1956*	*1.9424*	*1.1450*
% Fear	4.7720	4.5114	*1.9371*	*1.0632*
% Disgust	*1.8207^d^*	*1.2506*	*1.9520*	*1.1380*
% Anger	*1.9003*	4.6179	*1.8858^d^*	*0.6834*
% Anticipation	*1.9060*	*1.2348*	*1.9454*	*1.1088*
Best predictors: joy (%), negative sentiment (%), surprise (%), trust (%)	2.7323	33.5446	5.1538	*0.6816^d^*

^a^ARIMA: autoregressive integrated moving average.

^b^ARIMAX: autoregressive integrated moving average exogenous variable model.

^c^RMSE: root mean square error.

^d^Best-performing model for each metropolitan area.

### Effect of Models on Performance

To understand the effect of modeling choices on the usefulness of Twitter-derived features to improve COVID-19 vaccination rate predictions, we evaluated 2 additional models: one that used the *Syuzhet* package [45]—instead of BERT—to extract the same set of sentiments and emotions from tweets and then ARIMA/ARIMAX to predict COVID-19 vaccination rates; and another model that used BERT to extract sentiments and emotions from tweets and deep learning—a Temporal Fusion Transformer Model [46]—to predict COVID-19 vaccination rates, instead of ARIMA/ARIMAX. We confirmed that independently of the model selected, the same findings hold—the results of these models show that adding Twitter-based features to COVID-19 vaccination rates in predictive models improves most baselines, independently of the model and the city, albeit with higher RMSE than the ones shown in Tables 6 and 7. We have included descriptions, results, and a discussion of these other 2 models in Multimedia Appendix 2.

**Figure 2:**
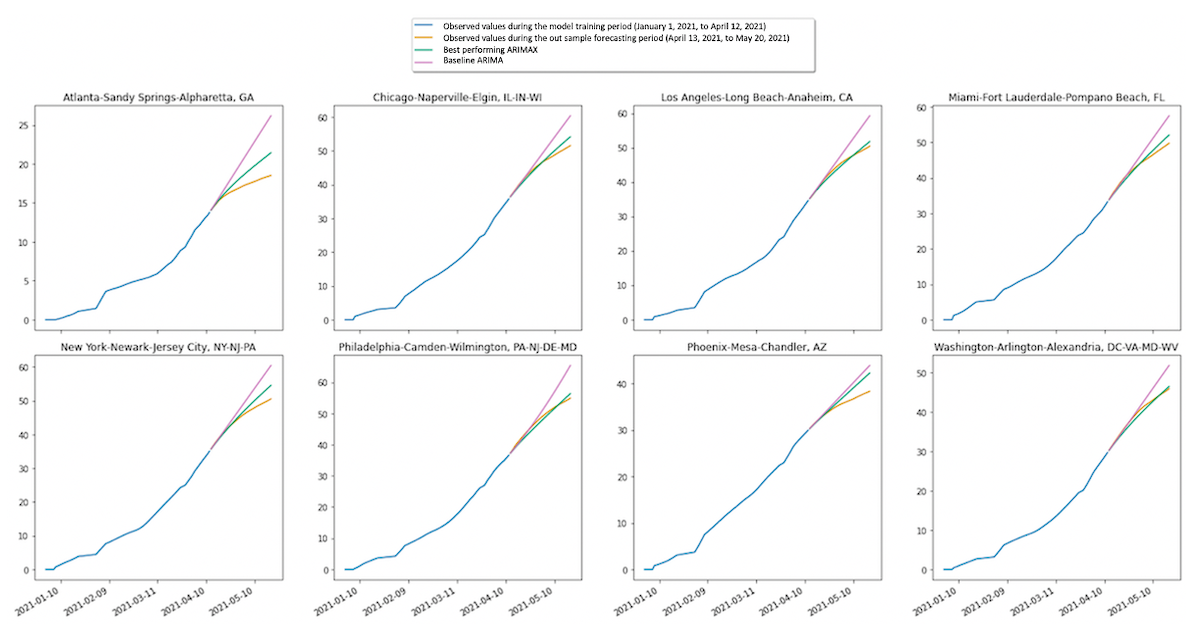
Predicted versus observed COVID-19 vaccination rates, January 1, 2021, to May 20, 2021. ARIMA: autoregressive integrated moving average; ARIMAX: autoregressive integrated moving average exogenous variable model.

Figure 2 illustrates the performance of the baseline ARIMA models and the best-performing ARIMAX models, compared to the observed values of the outcome variable during the “out-sample” forecasting period (April 13, 2021, to May 20, 2021). Across all metropolitan areas, the ARIMAX time series models with Twitter-derived features aligned more closely with the actual values of the vaccination rates compared to the baseline ARIMA model that relied on past historical vaccination data alone.

## Discussion

### Principal Findings

In this study, we sought to determine whether supplementing forecast models with COVID-19 vaccine attitudes found in tweets—modeled via sentiments and emotions—improves over baseline models that only use historical vaccination data. When evaluating model performance across all metropolitan areas, the addition of COVID-19 vaccine attitudes found in tweets resulted in improved model performance, as reflected by RMSE, when compared to baseline forecast models that did not include these features. Specifically, compared with the traditional ARIMA model with vaccination data alone, ARIMAX models with the predictions of both historical vaccination data and COVID-19 vaccine attitudes found in tweets reduced RMSE by as much as 83%. We were able to replicate similar findings across various modeling choices, including the *Syuzhet* package to extract sentiments and emotions, instead of BERT, and deep learning (temporal fusion transformer model) to predict COVID-19 vaccination rates, instead of ARIMA/ARIMAX.

### Study Findings in Context

The ongoing COVID-19 pandemic emphasizes the need for innovative approaches to public health surveillance. The global public health community has monitored the COVID-19 pandemic by tracking case counts, hospitalizations, deaths, and vaccinations. For the United States, these data sets are publicly available. Forecasting case counts and vaccination rates using existing historical data has been a key approach in COVID-19 surveillance efforts [47]. Previous forecast models for predicting vaccine uptake rate relied on traditional ARIMA methods, where historical data were used to predict future rates [48]. However, social media data sources, such as Twitter, reveal society’s attitudes toward the pandemic and current vaccination efforts on a real-time basis. This provides an opportunity for a large volume of raw and uncensored data related to vaccine attitudes, across various geographic locations, to be leveraged for disease surveillance, which can subsequently be used to supplement and improve existing models.

The findings of this study suggest that attitudes extracted from Twitter data can be added to existing forecast models for monitoring vaccination uptake across various metropolitan areas. In certain metropolitan areas, the mere volume of tweets and users engaged in vaccine-related conversations improved model performance when compared to baseline models. These results echo the findings in the study by Maugeri et al [33], which revealed another social media source, Google Trends data, improved the prediction of COVID-19 vaccination uptake in Italy when compared to baseline models. In this study, Google Trends data were represented as the relative search volume for each vaccine-related keyword. Another similar study developed a framework for predicting vaccination rates in the United States based on traditional clinical data and web search queries [49]. The results of this study also revealed the ability for online networks to predict societal willingness to receive vaccinations. Specifically, the authors similarly found improvement in model performance as in this study—with a reduction in RMSE of 9.1%.

Although few studies sought to supplement current vaccine models with social media data, to our knowledge, there are no studies that go beyond the mere volume of relevant Twitter data and factor in the sentiment and emotion of vaccine-related conversations. Over the course of the pandemic, some states experienced low vaccination rates despite comprehensive vaccine roll out programs. In these cases, it is important to consider the public’s emotions and sentiments toward vaccines. This study contributes to the literature by evaluating the ability for sentiments and emotions related to the COVID-19 vaccine to predict vaccine uptake. Specifically, the results show an improvement in model performance across metropolitan areas when models were supplemented with the percentage of tweets expressing anger, fear, joy, positive sentiment, or neutral sentiment. A study conducted by Alegado and Tumibay [48] examined the association between sentiments and emotions found in tweets and vaccine uptake via regression coefficient analysis. This study showed similar insights—tweets expressing fear, sadness, and anger appeared to be significantly associated with vaccination rates.

The results of this study have several implications for the present COVID-19 response. Public health experts now argue that the traditional concept of herd immunity may not apply to COVID-19 [2]. Instead, the focus is to increase vaccination uptake to substantially control community spread, without the societal disruptions caused by the virus [3]. Accurately forecasting vaccination uptake allows policy makers and researchers to evaluate how close we are to achieving normalcy again. Additionally, similar algorithms allow public health practitioners to better anticipate vaccine uptake behaviors and therefore develop targeted policies. As the global community builds toward achieving herd immunity, researchers should also “listen” to the vaccine conversation on social media—monitoring misconceptions and misinformation and implementing targeted vaccine education campaigns that address these misconceptions. Although the COVID-19 pandemic appears to be improving, the present framework can also be used to improve vaccine forecast models for future pandemics.

### Limitations and Future Work

It is important to note that this study has some limitations. The study period was limited to the first half of 2021. However, vaccines were not yet available to most of the US adult population until April 2021. Therefore, the study period did not capture the height of vaccination efforts. Another limitation is that as the COVID-19 pandemic evolves, vaccine related keywords may change, requiring frequent updating of the model. Future work may involve the use of topic modeling to capture the general themes surrounding the COVID-19 pandemic.

Another limitation is related to the geographic scope of this study. This study only focused on forecasting vaccine uptake in the United States. However, it is important to note that vaccination efforts must be addressed on a global scale, not just domestically, for normalcy to be attained. Future work should consider collecting tweets and vaccination data from other countries to see if similar models improve vaccine forecasts globally. Additionally, this study only examined tweets posted in the English language. Limiting the study to the collection of Tweets only in the English language poses a limitation as it may overlook valuable insights and perspectives expressed in other languages. This exclusion could lead to a biased understanding of sentiments and emotions, potentially missing out on crucial data from non–English-speaking populations. Language barriers may hinder the study's generalizability and restrict the representation of diverse cultural contexts. Future work should involve the use of sentiment and emotion classifiers that include lexicons in other languages.

### Conclusions

Researchers have found that the internet and social media both play a role in shaping personal or parental choices about vaccinations. Although few previous studies have developed forecast models for COVID-19 vaccination rates in the United States, to our knowledge, there are no studies that aim to factor in the real-time vaccination attitudes present on Twitter. This study suggests the benefits of using the linguistic constructs found in tweets to improve predictions of the COVID-19 vaccination rate. In this study, we found that supplementing baseline forecast models with both historical vaccination data and COVID-19 vaccine attitudes found in tweets reduced RMSE by as much as 83%. Developing a predictive tool for vaccination uptake in the United States will empower public health researchers and decision makers to design targeted vaccination campaigns in hopes of achieving the vaccination threshold required for widespread population protection.

## Appendix

Multimedia Appendix 1 COVID-19 vaccine keywords.

Multimedia Appendix 2 Alternative prediction models.

## Abbreviations

ARIMA: autoregressive integrated moving average
ARIMAX: autoregressive integrated moving average exogenous variable model
BERT: bidirectional encoder representation from transformer
RMSE: root mean square error
