# Supplementary material for: Using COVID-19 Vaccine Attitudes on Twitter to Improve Vaccine Uptake Forecast Models in the United States: Infodemiology Study of Tweets
Source: JMIR Infodemiology. 2023 Aug 21;3:e43703. doi: 10.2196/43703 (PMC10477926; doi:10.2196/43703)
Supplement: Multimedia Appendix 1 [file infodemiology_v3i1e43703_app1.docx]

**Multimedia Appendix 1.** COVID-19 vaccine keywords.

| pfizer |
| --- |
| vaccine |
| moderna |
| dose |
| doses |
| vaccines |
| johnson and johnson |
| johnson johnson |
| johnson & johnson |
| johnsonandjohnson |
| johnson&johnson |
| johnsonjohnson |
| shot |
