# Supplementary material for: Using COVID-19 Vaccine Attitudes on Twitter to Improve Vaccine Uptake Forecast Models in the United States: Infodemiology Study of Tweets
Source: JMIR Infodemiology. 2023 Aug 21;3:e43703. doi: 10.2196/43703 (PMC10477926; doi:10.2196/43703)
Supplement: Multimedia Appendix 2 [file infodemiology_v3i1e43703_app2.docx]

**Multimedia Appendix 2.** Alternative prediction models**.**

## 1.1 Syuzhet with ARIMA/ARIMAX

To capture the sentiments and emotions found in COVID-19 vaccine-related tweets, a sentiment and emotion analysis of all tweets was conducted using the NRC lexicon from the Syuzhet package in Python [1]. The NRC lexicon, developed by Saif Mohammad, contains a list of manually labeled English words and their associations with negative and positive sentiments and common human emotions, such as trust, fear, sadness, surprise, and disgust [2]. The Syuzhet package applies the NRC lexicon by independently evaluating and rating each word or expression within a tweet [3] . The get_nrc_sentiment function was applied to all tweets to calculate the valence of eight different emotions (fear, joy, anticipation, anger, disgust, sadness, surprise, trust), along with overall positive and negative sentiment. The percentages of the eight emotions, along with the percentage of positive, neutral, and negative sentiments were calculated at the metropolitan level. The ARIMA/ARIMAX prediction models are developed using the same approach described in the main manuscript, but with sentiments and emotions extracted using Syuzhet. Model performance is shown in Table S1 and Figure S1.

**Table S1.** Model performance (RMSE) of the models that used the Syuzhet package in Python to extract sentiments and emotions and ARIMA/ARIMAX to predict COVID-19 vaccination rates. Models that performed better than the baseline ARIMA are bolded. The best performing model for each metropolitan area is marked by an asterisk (*).

| **Variables** | **Atlanta-Sandy Springs-Alpharetta, GA** | **Chicago-Naperville-Elgin, IL-IN-WI** | **Los Angeles-Long Beach-Anaheim, CA** | **Miami-Fort Lauderdale-Pompano Beach, FL** |
| --- | --- | --- | --- | --- |
| (Baseline) % of individuals who have been administered at least one vaccine dose (7 day rolling average) | 4.08554185 | 4.21821418 | 4.14730143 | 3.7108186 |
| Number of users per 100,000 population | **1.636322** | **1.390199** | **0.719796** | **1.299161** |
| Number of tweets per 100,000 population | **1.643638** | **1.364628** | **0.713101** | **1.351403** |
| Average favorites | **2.117598** | **4.207971** | **0.707754** | 4.004478 |
| Average retweets | 5.354489 | **1.241377** | **4.13559** | **1.406216** |
| % Positive Sentiment | **1.59097** | **1.329552** | **0.725998** | **0.739024*** |
| % Negative Sentiment | **1.574885** | **1.282263** | **0.709669** | **3.705101** |
| % Neutral Sentiment | **1.598352** | **1.225369** | **0.728478** | **1.067679** |
| % Trust | **1.634385** | **1.330901** | **0.705849** | **1.143485** |
| % Surprise | **1.567964** | **1.307367** | **0.706967** | 3.76972 |
| % Sadness | **1.553468** | **1.306249** | **0.700871*** | **1.034181** |
| % Joy | **1.523055*** | **1.268508** | **0.713668** | **1.201604** |
| % Fear | **1.550904** | **1.341739** | **0.72593** | **3.645123** |
| % Disgust | **1.575286** | **1.246528** | **0.706552** | **1.131289** |
| % Anticipation | **1.660202** | **1.304346** | **0.707404** | **1.258046** |
| % Anger | **4.053352** | **1.335557** | **0.7114** | **1.061867** |
| Best Predictors (% Joy, % Negative Sentiment, % Surprise, % Trust) | **1.558327373** | **0.743538748*** | **1.081685146** | **0.7711874** |
|  | **New York-Newark-Jersey City, NY-NJ-PA** | **Philadelphia-Camden-Wilmington, PA-NJ-DE-MD** | **Phoenix-Mesa-Chandler, AZ** | **Washington-Arlington-Alexandria, DC-VA-MD-WV** |
| (Baseline) % of individuals who have been administered at least one vaccine dose (7 day rolling average) | 4.76859187 | 5.52806379 | 2.77431054 | 2.58293614 |
| Number of users per 100,000 population | **1.845547** | **1.251691** | **1.998467** | **1.155822** |
| Number of tweets per 100,000 population | **1.833835** | **1.273346** | **1.976664** | **1.126215** |
| Average favorites | **4.756388** | 5.607816 | **1.940556** | **0.756991*** |
| Average retweets | **1.88377** | **0.91997*** | **2.772791** | **0.757008** |
| % Positive Sentiment | **1.790318** | **1.189359** | **1.971136** | **1.123634** |
| % Negative Sentiment | **4.755769** | **1.289902** | **2.027737** | 2.718688 |
| % Neutral Sentiment | **1.736168*** | **2.659972** | **1.940593** | **0.936273** |
| % Trust | **1.976131** | **1.26753** | **1.926827** | **1.481591** |
| % Surprise | **1.875066** | **2.642564** | **1.931609** | **1.142052** |
| % Sadness | **1.891604** | **2.570778** | **1.926981** | **2.564708** |
| % Joy | **1.825016** | **1.673547** | **1.933832** | **2.473224** |
| % Fear | **1.930926** | **1.250402** | **1.937204** | **1.135616** |
| % Disgust | **1.891015** | **1.676407** | **1.94021** | **2.579913** |
| % Anger | **1.898955** | **1.247587** | **1.943948** | 2.738729 |
| % Anticipation | **1.910527** | **1.67782** | **1.886751*** | **2.560481** |
| Best Predictors (% Joy, % Neutral Sentiment, % Fear, % Sadness, % Positive Sentiment) | **1.875641904** | **1.778891549** | **1.955301651** | **1.336705367** |

**
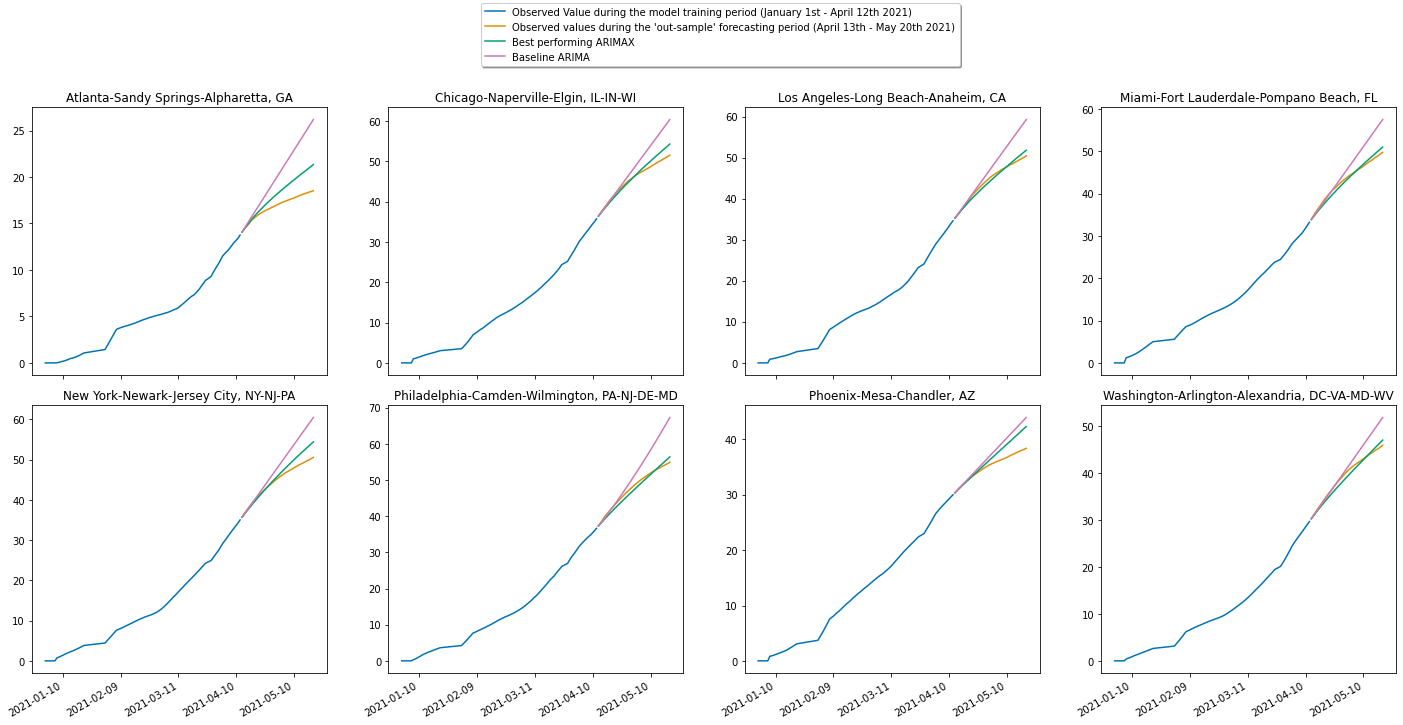
Figure S1.** Predicted vs observed COVID-19 vaccination rates for the models that used the Syuzhet package in Python to extract sentiments and emotions and ARIMA/ARIMAX to predict COVID-19 vaccination rates, January 1 – May 20, 2021.

## 1.2 BERT with Deep Learning (TFT)

For this model, we used BERT to extract sentiments and emotions as described in the paper. To predict COVID-19 vaccination rates using Twitter-based features, sentiments, and emotions, we used a state-of-the-art deep learning-based forecasting model: Temporal Fusion Transformer Model (TFT) implemented in PyTorch Forecasting [4], [5] . The TFT architecture consists of LSTM encoders, gated residual networks, temporal attention head, and feedforward layers to perform autoregressive tasks in an interpretable manner. The TFT model allows for incorporation of time-dependent parameters, static parameters, as well as categorical exogenous variables in the forecasting tasks. We use an encoder length of 14, learning rate of 0.02 and 1 attention head to train the model with the Quantile Loss. To prevent overfitting, we use the Early Stop mechanism by tracking the training and validation metrics. We train TFT models using only past vaccination rates as a baseline or adding to past vaccination rates and one or more sentiment and emotion features extracted using the TweetNLP BERT model, as described in the main manuscript. Model performance is shown in Table S2 and Figure S2.

**Table S2.** Model performance (RMSE) of the models that used Bidirectional Encoder Representation from Transformer (BERT) to extract sentiments and emotions and deep learning (Temporal Fusion Transformer Model, TFT) to predict COVID-19 vaccination rates. Models that performed better than the baseline ARIMA are bolded. The best performing model for each metropolitan area is marked by an asterisk (*).

| **Variables** | **Atlanta-Sandy Springs-Alpharetta, GA** | **Chicago-Naperville-Elgin, IL-IN-WI** | **Los Angeles-Long Beach-Anaheim, CA** | **Miami-Fort Lauderdale-Pompano Beach, FL** |
| --- | --- | --- | --- | --- |
| (Baseline) % of individuals who have been administered at least one vaccine dose (7 day rolling average) | 7.650367 | 18.30835 | 16.75044 | 15.34508 |
| Number of users per 100,000 population | **3.024083*** | **7.70336** | **7.556329** | **6.602613** |
| Number of tweets per 100,000 population | **5.000582** | **6.424729*** | **6.345813** | **6.242342** |
| Average favorites | 13.95015 | **12.91989** | **11.12095** | **9.464826** |
| Average retweets | 10.77635 | **7.601187** | **7.064871** | **6.39494** |
| % Positive Sentiment | 7.994034 | **10.55065** | **9.447006** | **8.541215** |
| % Negative Sentiment | 12.69921 | **9.313119** | **8.317041** | **7.525572** |
| % Neutral Sentiment | 8.384473 | **16.40825** | **14.54201** | **13.65173** |
| % Trust | 12.05347 | **6.722482** | **6.226737** | **5.635925** |
| % Surprise | **6.075604** | **10.13256** | **8.96493** | **7.932856** |
| % Sadness | 18.50355 | **6.967054** | **6.198871*** | **5.478134** |
| % Joy | 16.74274 | **7.016842** | **6.777584** | **5.423577*** |
| % Fear | **5.458589** | **10.47826** | **9.545059** | **9.94082** |
| % Disgust | **12.18567** | 33.66411 | 32.38037 | 31.53346 |
| % Anger | **5.780242** | **11.56145** | **10.37887** | **7.116512** |
| % Anticipation | **4.926801** | **10.97355** | **9.655929** | **8.46081** |
| Best Predictors (% Joy, % Negative Sentiment, % Surprise, % Trust) | **3.875413** | **7.845341** | **7.433683** | **6.431036** |
|  | **New York-Newark-Jersey City, NY-NJ-PA** | **Philadelphia-Camden-Wilmington, PA-NJ-DE-MD** | **Phoenix-Mesa-Chandler, AZ** | **Washington-Arlington-Alexandria, DC-VA-MD-WV** |
| (Baseline) % of individuals who have been administered at least one vaccine dose (7 day rolling average) | 17.32175 | 20.20366 | 5.665681 | 9.373214 |
| Number of users per 100,000 population | **7.281109** | **8.166649** | **5.119769** | **8.340237** |
| Number of tweets per 100,000 population | **6.242421*** | **6.571311*** | 6.450185 | **7.136421** |
| Average favorites | **11.45313** | **15.82283** | **5.566754** | **7.989687** |
| Average retweets | **7.121293** | **8.373845** | **4.557721** | **6.463762** |
| % Positive Sentiment | **9.827587** | **11.83335** | **4.327697** | **5.551282** |
| % Negative Sentiment | **8.578468** | **10.89533** | **3.534085** | **4.530615*** |
| % Neutral Sentiment | **15.83872** | **18.54506** | **7.925471** | **9.014196** |
| % Trust | **6.30314** | **7.494084** | **4.067691** | **6.083385** |
| % Surprise | **9.381115** | **11.5042** | **3.727259** | **4.877216** |
| % Sadness | **6.383041** | **7.97202** | **3.101509** | **5.320641** |
| % Joy | **6.676277** | **7.461914** | 5.781122 | **7.575409** |
| % Fear | **9.638756** | **12.37062** | 10.18158 | **7.470041** |
| % Disgust | 32.93481 | 34.93579 | 27.31276 | 28.0164 |
| % Anger | **10.76204** | **12.18232** | **3.462406** | **5.915105** |
| % Anticipation | **10.06743** | **12.32247** | **3.736074** | **4.845652** |
| Best Predictors (% Joy, % Negative Sentiment, % Surprise, % Trust) | **7.376978** | **9.060766** | **4.461446** | **6.237017** |


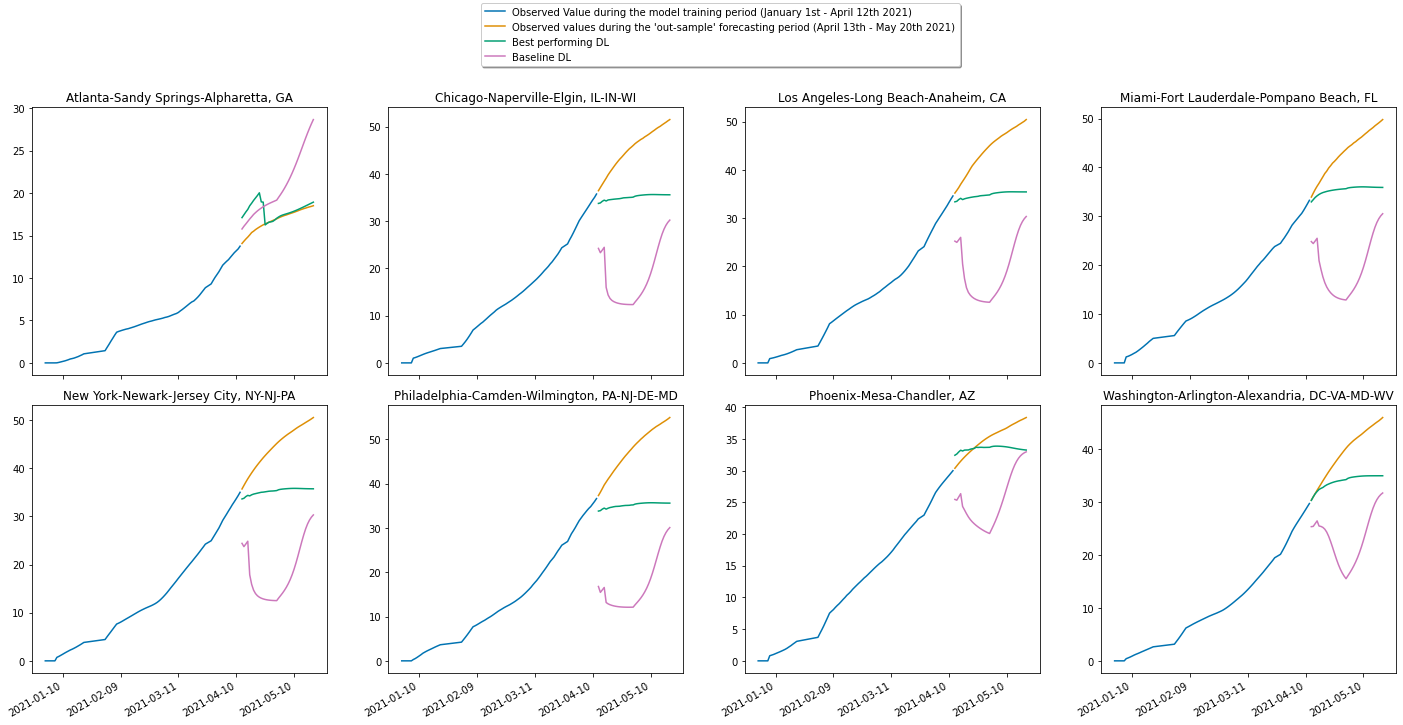
**Figure S2.** Predicted vs observed COVID-19 vaccination rates for the models that used Bidirectional Encoder Representation from Transformer (BERT) to extract sentiments and emotions and deep learning (Temporal Fusion Transformer Model, TFT) to predict COVID-19 vaccination rates, January 1 – May 20, 2021.

## 1.3 Model Performance

Overall, the Syuzhet+ARIMAX and BERT+TFT presented in this Appendix in Sections 1.1 and 1.2 confirm the findings described in the main manuscript: that independently of the model selected and the city, adding Twitter-based features to COVID-19 vaccination rates predictive models improves most baselines. Our results show that almost all sentiments, emotions, or Twitter user characteristics improve the errors of the baselines that exclusively use COVID-19 vaccination rates, with on average between 12 and 15 features (out of the total 16) improving the baselines for each city in our study. This confirms our hypothesis that Twitter features provide information about human attitudes towards the COVID-19 vaccine and help improve COVID-19 vaccination rate predictions. A high-level comparison of Tables S1 and S2 with Table 6 in the main manuscript shows that Syuzhet+ARIMAX and BERT+ARIMAX share similar RMSE performance, with BERT sentiments and emotions working slightly better; and that BERT+TFT models show a lower performance with higher RMSE than their ARIMA/ARIMAX counterpart.

More in depth, our results show average errors per sentiment for the BERT+ARIMAX and Syuzhet+ARIMAX models in the (1.3-3.3) range, with 6 sentiments performing better, on average across cities, for the BERT+ARIMAX models and 5 sentiments performing better, on average, for the Syuzhet+ARIMAX approach. Past work has shown that Syuzhet can outperform BERT sentiment analysis in short texts like tweets [6], and we argue that our results possibly confirm this finding.

On the other hand, we observe that BERT+TFT models have, on average, higher RMSE values compared to the Syuzhet/BERT+ARIMAX models, with average errors per sentiment in the (6.30-13.03) range and with disgust associated with average errors of up to 29. We attribute these differences in performance to the size of the dataset per metropolitan area, with 128 points for training and testing. We believe that one of the challenges of this forecasting problem is the small sample size, which constitutes a challenge for “data-hungry” deep learning-based forecasting approaches [7]. Future work will investigate BERT+TFT’s predictive performance with larger datasets for both COVID-19 vaccination rates and Twitter-based features, including sentiments and emotions.

# References

[1] M. Jockers, “syuzhet: Extracts Sentiment and Sentiment-Derived Plot Arcs from Text.” Nov. 24, 2020. Accessed: Jun. 08, 2023. [Online]. Available: https://cran.r-project.org/web/packages/syuzhet/

[2] “NRC Emotion Lexicon.” http://saifmohammad.com/WebPages/NRC-Emotion-Lexicon.htm (accessed Jan. 13, 2022).

[3] M. A. Kausar, A. Soosaimanickam, and M. Nasar, “Public Sentiment Analysis on Twitter Data during COVID-19 Outbreak,” *International Journal of Advanced Computer Science and Applications (IJACSA)*, vol. 12, no. 2, Art. no. 2, 58/01 2021, doi: 10.14569/IJACSA.2021.0120252.

[4] “Interpretable Deep Learning for Time Series Forecasting,” Dec. 13, 2021. https://ai.googleblog.com/2021/12/interpretable-deep-learning-for-time.html (accessed Jun. 08, 2023).

[5] J. Beitner, “Installation.” Jun. 08, 2023. Accessed: Jun. 08, 2023. [Online]. Available: https://github.com/jdb78/pytorch-forecasting

[6] A. Kotelnikova, D. Paschenko, K. Bochenina, and E. Kotelnikov, “Lexicon-based Methods vs. BERT for Text Sentiment Analysis.” arXiv, Nov. 19, 2021. doi: 10.48550/arXiv.2111.10097.

[7] A. Adadi, “A survey on data‐efficient algorithms in big data era,” *Journal of Big Data*, vol. 8, no. 1, p. 24, Jan. 2021, doi: 10.1186/s40537-021-00419-9.
